# Physical activity, sleep disorders, and type of work in the prevention of cognitive function decline in patients with hypertension

**DOI:** 10.1186/s12889-023-17343-7

**Published:** 2023-12-06

**Authors:** Mengdi Zhang, Huachen Jiao, Cong Wang, Ying Qu, Shunxin Lv, Dongsheng Zhao, Xia Zhong

**Affiliations:** 1 The First Clinical Medical College, Shandong University of Traditional Chinese Medicine, Jinan, China; 2 Department of Cardiology, Affiliated Hospital of Shandong University of Traditional Chinese Medicine, No. 42, Wenhua West Road, Lixia District, Jinan, Shandong China; 3 College of Traditional Chinese Medicine, Shandong University of Traditional Chinese Medicine, Jinan, China

**Keywords:** Hypertension, Sleep disorders, Cognitive function decline, Physical activity, Type of work

## Abstract

**Background:**

Hypertensive patients are likelier to have cognitive function decline (CFD). This study aimed to explore physical activity level, sleep disorders, and type of work that influenced intervention effects on cognitive function decline in hypertensive patients and to establish a decision tree model to analyze their predictive significance on the incidence of CFD in hypertensive patients.

**Methods:**

This cross-sectional study recruited patients with essential hypertension from several hospitals in Shandong Province from May 2022 to December 2022. Subject exclusion criteria included individuals diagnosed with congestive heart failure, valvular heart disease, cardiac surgery, hepatic and renal dysfunction, and malignancy. Recruitment is through multiple channels such as hospital medical and surgical outpatient clinics, wards, and health examination centers. Cognitive function was assessed using the Mini-Mental State Examination (MMSE), and sleep quality was assessed using the Pittsburgh Sleep Quality Index (PSQI). Moreover, we obtained information on the patients' type of work through a questionnaire and their level of physical activity through the International Physical Activity Questionnaire (IPAQ).

**Results:**

The logistic regression analysis results indicate that sleep disorder is a significant risk factor for CFD in hypertension patients(OR:1.85, 95%CI:[1.16,2.94]), mental workers(OR:0.12, 95%CI: [0.04,0.37]) and those who perform both manual and mental workers(OR: 0.5, 95%CI: [0.29,0.86]) exhibit protective effects against CFD. Compared to low-intensity, moderate physical activity(OR: 0.53, 95%CI: [0.32,0.87]) and high-intensity physical activity(OR: 0.26, 95%CI: [0.12,0.58]) protects against CFD in hypertension patients. The importance of predictors in the decision tree model was ranked as follows: physical activity level (54%), type of work (27%), and sleep disorders (19%). The area under the ROC curves the decision tree model predicted was 0.72 [95% CI: 0.68 to 0.76].

**Conclusion:**

Moderate and high-intensity physical activity may reduce the risk of developing CFD in hypertensive patients. Sleep disorders is a risk factor for CFD in hypertensive patients. Hypertensive patients who engage in mental work and high-intensity physical activity effectively mitigate the onset of CFD in hypertensive patients.

## Introduction

Cognitive function decline (CFD) is a transitional stage between normal cognitive function and dementia [1], and its primary clinical manifestations are cognitive impairment, memory loss, and retention of daily living ability [2]. According to the epidemiological survey in China in 2020, the prevalence rate of dementia among people aged 60 and above was 6.04%, with 15.7 million people, among which there were 9.83 million alzheimer's disease(AD) and 38.77 million CFD [3]. As with CFD, the risk for developing hypertension increases with age. According to the Guidelines for the pharmacological treatment of Hypertension in Adults, 1.4 billion people worldwide have hypertension in 2021 [4]. Approximately 70% of people older than 70 years of age worldwide have hypertension, and the prevalence increases with age [5]. According to Petersen et al. (2018), the prevalence of CFD increases with age and ranges from 6.7% in people aged 60 to 64 to 25.2% in people aged 80 to 84 [6]. Hypertension is increasingly recognized as a risk factor for cognitive impairment [7]. According to the China Nationwide Hypertension Survey (2012–2015), 23.2% of Chinese individuals aged 18 and older had hypertension (244.5 million people) [8]. A 2015–2018 Chinese epidemiological survey published in 2020 found that the risk of developing CFD was 1.62 times higher in hypertensive patients than in non-hypertensive patients [3]. Hypertension is recognised as a major risk factor for cognitive impairment, potentially increasing the risk of vascular dementia and Alzheimer's disease [7].The pathogenesis of hypertension-related CFD is complex and incompletely understood because of aging populations and the growing prevalence of hypertension. A growing body of evidence shows that the onset and development of CFD result from the interaction of several factors. In a prior study, researchers discovered and ranked risk factors associated with CFD. The study underscored living alone, smoking, and a high-fat diet as pivotal factors influencing the onset of CFD [9].

Previous research has suggested that several factors, including age, hypertension, diabetes, LDL-C, blood homocysteine, walking speed, social activities, and risk of falling, contribute significantly to the development of cognitive function decline. There is a strong association between hypertension, diabetes, and cerebrovascular disease with cognitive function decline. There is also an association between cognitive function and dietary habits and physical activity. However, most studies have only examined the relationship between several factors and CFD and have yet to explain further the interaction between different factors that affect CFD [10–15]. Decision trees are a potent and uncomplicated machine learning model. They have many applications in public health and health behavior research, assisting in subgroup identification based on decision-making processes. Yong et al. utilized this method to distinguish high-probability and low-probability quitters among smokers, achieving moderate accuracy rates ranging from 0.62 to 0.71 [16–20]. As one of the most widely used classification and prediction methods, the Decision Tree algorithm can comprehensively analyze various factors affecting CFD. Moreover, the decision tree approach has been applied to forecast mild cognitive decline in senior citizens [9]. Wang explored the benefits of using decision tree modeling in clinical and disease management through clinical studies, focusing on ease of understanding and interpretation. The results show that decision trees are effective in predicting CFD, with an AUC of 0.83[95% CI: 0.80 to 0.86] on the ROC curve. However, there are limitations to this study. The lack of information on lifestyle factors, such as the duration, intensity, and type of physical activity, may affect the usefulness and accuracy of the model. Additionally, not enough information on the type and frequency of tea consumption may impact the model's accuracy. Furthermore, increasing the sample size is expected to improve the consistency and usefulness of the model and enhance the credibility of the study. Nevertheless, we scarcely identified any decision tree-based model to anticipate mild cognitive debilitation in people with hypertension. Therefore, this paper attempts to introduce the Chi-squared Automatic Interaction Detection (CHAID) decision tree algorithm into the analysis of influencing factors of CFD in hypertensive patients to conduct rapid screening of cognitive impairment with maximum accuracy to intervene or delay its occurrence and development.

## Methods

### Study design and population

In this study, a stratified cluster sampling method was employed to select five prefecture-level cities in Shandong Province, namely Jinan, Yantai, Weifang, Dongying, and Jining. Subsequently, eight hospitals were randomly selected from these cities. The primary objective of the research was to investigate hypertensive patients in these hospitals, and the recruitment process took place from May 2022 to December 2022. Individuals aged 40 and over who were diagnosed with essential hypertension were recruited to participate in this study, and CFD diagnoses were made by two experienced physicians. Patients with congestive heart failure, valvular heart disease, cardiac surgery, liver and kidney dysfunction, and malignancy were excluded from the study. The Medical Research Ethics Committee of the Affiliated Hospital of Shandong University of Traditional Chinese Medicine approved this study (( 2020) Lunshen No. ( 011) -KY|).Informed consent was obtained from all participants.

### Data collection

Structured questionnaires were administered to collect information on confounding variables related to cognitive function decline, including sociodemographic characteristics, educational level, type of work, lifestyle, medical history, and current medications. We classified occupation types into three categories: mental labor (professionals, government), both manual and brain labor (skilled workers, service providers, merchants), and manual labor (farmer, factory worker, manufacturing, transportation) [21, 22]. Sleep quality was measured using the Pittsburgh Sleep Quality Index (PSQI), and daily physical activity was assessed using the International Physical Activity Questionnaire (IPAQ). Data were stored on a secure server with access restricted to authorized personnel.

Professional nurses measured blood pressure in a quiet environment and performed anthropometric measurements such as height, weight, and waist circumference on subjects wearing light clothing and no shoes. Fasting blood glucose (FBG), triglycerides (TG), total cholesterol (TC), low-density lipoprotein (LDL), and serum creatinine (SCr) were measured in all subjects after overnight fasting. The lipid accumulation product (LAP) index was calculated according to the following equations: LAP = [waist circumference (WC) (cm) – 65] × TG (mmol/L) for males and [WC (cm) – 58] × TG (mmol/L) for females (65 and 58 represent the estimated minimum WC values for men and women, respectively) [23].

### Cognitive function assessment

Cognitive function was assessed in this study using the MMSE. The MMSE demonstrates significant sensitivity to cognitive impairment in construct validity tests. A correlation between the Blessed Information-Memory-Concentration test, which assesses cognitive functioning, and the MMSE ranged from 0.70 to 0.90. According to Foreman [24], the MMSE has a reliability ranging from 0.77 to 0.99. Folstein [25] developed the MMSE, and later modified by Li has a Cronbach's alpha of 0.97in its Chinese version [26]. The MMSE assesses five cognitive domains: orientation, attention, memory, language, and visuospatial ability. It is a highly sensitive cognitive screening tool that detects and discriminates patients with cognitive impairment from cognitively normal individuals. The MMSE has a total score of 30, with higher scores indicating better cognitive function [27]. Subjects who obtained scores lower than 27 on the MMSE were diagnosed with cognitive function decline [28, 29].

### Anthropometric measurements of adiposity

Patients’ weight and height were measured while they were wearing light clothing. Body mass index (BMI) was calculated as weight (kg) divided by the square of height (m^2^). Obesity (BMI > 30) or not [16] was determined by BMI [30]. An experienced nurse measured waist circumference (WC) at the midway point between the lower edge of the rib cage and the iliac crest. The measurement was taken twice, and the average value was recorded [31].

### Definition of hypertension

Blood pressure readings were obtained from all participants during a single visit. Systolic blood pressure (SBP) and diastolic blood pressure (DBP) were measured every 2 min, and the average of three consecutive readings was calculated. Hypertension was defined as SBP of 140 mmHg or higher, DBP of 90 mmHg or higher, or the use of antihypertensive medication [27].

### Pittsburgh sleep quality index

A total of 18 items were scored, including seven components: subjective sleep quality, sleep onset latency, sleep duration, sleep efficiency, sleep disturbance, hypnotic medication, and daytime dysfunction. Total scores range from 0 to 21, with higher scores indicating worse sleep quality. In this study, PSQI > 7 was defined as a sleep disorder [32].

### Physical activity levels

The International Physical Activity Questionnaire (IPAQ) [33–35] assessed physical activity (PA) levels. By reporting the duration of vigorous activity, moderate activity, and walking the previous week, participants' MET values were estimated by combining various PA types using corresponding coefficients. One MET corresponds to the energy expended by sitting quietly. Eight is the coefficient of the compendium average MET for vigorous activity. For moderate physical activity, the coefficients were four. For walking as a physical activity, the coefficients were 3.3. Physical activity was categorized into low, moderate, and high. PA must satisfy one of the following two requirements to be classified as high: (1) At least 1,500 MET-min/week of vigorous exercise, performed at least three days each week. (2) Walking, strenuous activity, or any combination of the three for at least five days per week, collecting at least 3,000 MET-min each week; One of the following four requirements must be met for PA to be classified as moderate: (1) Perform intense movement for at least 20 min three days a week. (2) Moderate exercise five days a week for at least 30 min. (3) Walking five or more days a week for at least 30 min each time. (4) A weekly average of at least 600 MET-min in total.

### Smoking and drinking status

Smoking was defined as: (1) having smoked fewer than 100 cigarettes, never a smoker; (2) having smoked more than 100 cigarettes and not currently smoking, former smoker; and (3) having smoked more than 100 cigarettes and currently smoking, current smoker [36]. In the analysis, passive smokers were not taken into account [37]. Drinking was defined as drinking alcohol on average every week for more than one year and still drinking at the time of the study. Participants were classified into current drinkers, nondrinkers, and ex‐drinkers [38].

### Statistical analysis

The statistical analysis was conducted using SPSS version 24.0. Quantitative data were presented as mean ± standard deviation (SD) or median (interquartile range), and group differences were compared using either the T test or the Mann–Whitney U test. Categorical variables were expressed as frequencies (percentages), and group differences were assessed using the chi-square test. Logistic regression models, both single-factor and multi-factor, were utilized to identify risk factors for cognitive decline, with cognitive decline serving as the dependent variable. A predetermined significance level of *P* < 0.05, using a two-tailed test, was employed to determine statistical significance. Logistic regression analysis was employed to identify the risk factors. The education level was categorized with Primary school or below as the reference group, the work type was categorized with manual work as the reference group, and the physical activity was categorized with light as the reference group. The decision tree model was constructed using the decision tree CHAID algorithm of IBM SPSS Modeler 18.0 software. To evaluate overfitting, the model was tested using the cross-validation method. The sampling consisted of ten groups, and the decision tree had a maximum depth of three. The parent node contained at least 50 samples, while the child nodes contained at least 20 samples. The split confidence level was set at 95%. ROC curves were generated utilizing MedCalc 19 software. The diagnostic performance of the decision tree models was assessed using both MedCalc V.19 and the Clinical Utility Index Calculator.

## Results

**Fig. 1:**
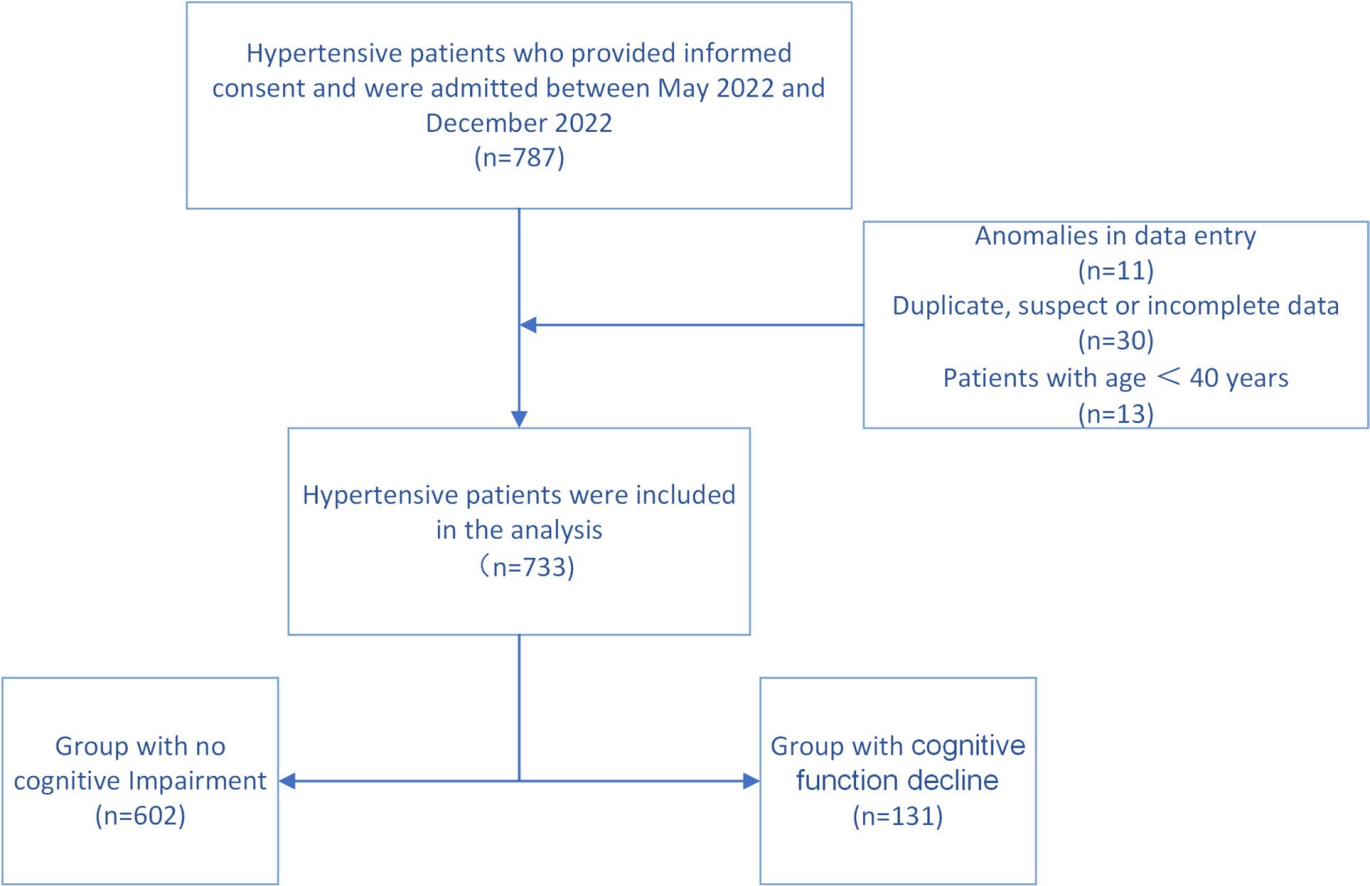
Flow chart of the study population

Figure 1 shows a flow chart describing the selection process of the participants in this study.

### Participants and baseline characteristics

Table 1 displays the demographic and clinical characteristics of the participants in the study. Out of the 733 hypertensive patients, 131 were diagnosed with CFD, with 63 males and 68 females. The remaining 602 patients did not show any cognitive impairment (NCI), with 293 males and 309 females. It was observed that the CFD group had a higher average age, which was significantly associated with cognitive decline (*p* < 0.01). Additionally, the CFD group exhibited higher levels of physical characteristics such as WC, BMI, left ventricular diameter (LVD), left atrial diameter (LAD), and right atrial diameter (RAD) (*p* < 0.05), while there was no difference between the two groups in terms of right ventricular diameter (RVD)(p > 0.05). Individuals with lower levels of education and those engaged in manual labor were found to be more susceptible to cognitive impairment (*p* < 0.05). In terms of lifestyle habits, the NCI group showed a higher prevalence of smoking (*p* < 0.05), while there was no difference in average monthly salt intake and alcohol consumption history between the two groups. Regarding medication, patients in the NCI group were more likely to use beta-blockers and angiotensin-converting enzyme inhibitors (ACEI)/angiotensin receptor blockers (ARB) (*p* < 0.05), with no statistical difference in the use of calcium channel blockers (CCBs) and diuretics. There was no difference between the two groups in terms of gender and marital status (*p* > 0.05). These findings suggest that cognitive decline in patients with CFD may be influenced by various factors, including older age, abnormal physical characteristics, lower levels of education, engagement in manual labor, and specific medication usage.

**Table 1:** Baseline characteristics of hypertensive patients with no cognitive impairment and cognitive function decline

Variables	NCI(*n* = 602)	CFD(*n* = 131)	*P*-value
Age, years	65.90 ± 10.49	76.07 ± 7.80	< 0.001^*^
Sex (Male), n (%)	293.00(48.67)	63.00(48.09)	0.904
Smoking, n (%)	92.00(15.28)	11.00(8.40)	0.040^*^
Drinking, n (%)	111.00(18.44)	18.00(13.74)	0.201
Marital status, n (%)			0.202
Married	589.00(97.84)	125.00(95.42)	
Unmarried, divorced or widowed	13.00(2.16)	6.00 (4.58)	
Educational level, n (%)			0.048^*^
Primary school or below	230(38.21)	65.00(49.61)	
Junior high school or senior high school	336.00(55.81)	61.00(46.56)	
University or above	36.00(5.98)	5.00(3.82)	
Type of work, n (%)			< 0.001^*^
Manual labor	295.00(49.00)	99.00(75.57)	
Mental labor	97.00(16.11)	4.00(3.05)	
Both manual and brain labor	210.00(34.88)	28.00(21.37)	
Obesity	269(44.7)	70(53.4)	0.069
Waist circumference, cm	82.78 ± 15.64	87.30 ± 12.65	< 0.001^*^
BMI, kg/m^2^	24.76 ± 3.26	25.38 ± 3.11	0.049^*^
MMSE score, points	29.24 ± 1.00	22.48 ± 3.63	< 0.001^*^
Average salt intake per month, g	300.00[180.00, 600.00]	270.00[180.00, 450.00]	0.051
Imaging parameters			
LVD (mm)	47.13 ± 6.57	49.02 ± 5.71	0.002^*^
RVD (mm)	21.91 ± 3.00	22.47 ± 3.87	0.066
LAD (mm)	36.39 ± 5.76	38.92 ± 6.55	< 0.001^*^
RAD (mm)	32.77 ± 5.60	34.91 ± 6.51	< 0.001^*^
Medication information, n (%)			
ACEI/ARBs use	394.00(65.45)	69.00(52.00)	0.006^*^
Beta-blockers use	125.00(20.76)	17.00(13.00)	0.041^*^
CCBs use	364.00(60.47)	81.00(61.83)	0.772
Diuretics use	113.00(18.77)	24.00(18.32)	0.905

Data are presented as mean ± SD, median [IQR] or n(%)

*Abbreviations*: *NCI* no cognitive impairment, *CFD* cognitive function decline, *BMI* body mass index, *MMSE* mini-mental state examination, *RAD* right atrial diameter, *LAD* left atrial diameter, *RVD* right ventricular diameter, *LVD* left ventricular diameter, *ACEI* angiotensin converting enzyme inhibitors, *ARB* angiotensin receptor blocker, *CCBs* calcium channel blockers

^*^Statistically significant value (*p* < 0.05)

As indicated in Table 2, individuals experiencing CFD exhibited a prolonged period of hypertension, elevated SBP and DBP, and increased levels of LAP and serum creatinine (*p* < 0.05). However, initial analyses revealed no statistically significant disparities between the two groups in relation to FBG, TG, TC, and LDL-C (*p* > 0.05).

**Table 2:** Blood Pressure and metabolism in hypertensive patients with no cognitive impairment and cognitive function decline

Variables	NCI(*n* = 602)	CFD(*n* = 131)	*P*-value
Duration of hypertension(m)	111.00[58.75, 179.00]	134.00[67.00, 219.00]	0.020^*^
SBP, mmHg	140.51 ± 11.79	144.59 ± 13.52	0.001^*^
DBP, mmHg	82.54 ± 10.17	84.62 ± 9.62	0.033^*^
LAP index	27.49[11.38, 48.59]	31.80[17.36, 53.13]	0.026^*^
Laboratory testing parameters			
FBG, mmol/L	5.76[5.20, 7.07]	5.94[5.26, 7.24]	0.255
TG, mmol/L	1.32[0.95, 1.94]	1.36[0.93, 1.77]	0.911
TC, mmol/L	4.58[3.76, 5.43]	4.45[3.42, 5.36]	0.216
LDL-C, mmol/L	2.67[2.03, 3.37]	2.54[1.88, 3.17]	0.131
SCr, μmoI/L	65.00[54.68, 78.00]	71.00[59.50, 87.00]	0.001^*^

*Abbreviations*: *NCI* no cognitive impairment, *CFD* cognitive function decline, *SBP* systolic blood pressure, *DBP* diastolic blood pressure, *LAP* lipid accumulation product, *FBG* fasting blood glucose, *TG* triglyceride, *TC* total cholesterol, *LDL-C* low-density lipoprotein cholesterol, *SCr* serum creatinine

^*^Statistically significant value (*p* < 0.05)

According to Table 3, individuals suffering from hypertension with CFD had worse sleep quality and appeared to be more prone to sleep disturbances (*P* < 0.01).

**Table 3:** Sleep status of hypertensive patients with no cognitive impairment and cognitive function decline

Variables	NCI(*n* = 602)	CFD(*n* = 131)	*P*-value
PSQI score, points	5.00[3.00, 8.00]	7.00[6.00, 9.00]	< 0.001^*^
sleep disorders	174(28.9)	62(47.3)	< 0.001^*^

^*^Statistically significant value (*p* < 0.05)

*Abbreviations*: *NCI* no cognitive impairment, *CFD* cognitive function decline, *PSQI* Pittsburgh sleep quality index

The physical activity level of each group is shown in Table 4. Hypertensive patients with vigorous physical activity levels had a lower risk of CFD than low and moderate physical activity levels(*p* < 0.01).

**Table 4:** Physical activity level of hypertensive patients with no cognitive impairment and cognitive function decline

Variables	NCI(*n* = 602)	CFD(*n* = 131)	*P*-value
Physical activity level, n (%)			< 0.001^*^
Light	113.00 (18.77)	50.00(38.17)	
Moderate	323.00 (53.65)	70.00(53.44)	
Vigorous	166.00 (27.57)	11.00(8.40)	

*Abbreviations*: *NCI* no cognitive impairment, *CFD* cognitive function decline

^*^Statistically significant value (*p* < 0.05)

### Multivariate regression analyses

The ORs for the multivariate model of cognitive function decline according to prespecified influencing factor are shown in Table 5. A binary logistic regression analysis revealed several factors contributing to CFD: age, work type, physical activity level, sleep disorders, ACEI/ARB usage, and Beta-blocker usage. Among these, age ≥ 65 years (OR: 8.68, 95% CI [3.64, 20.72], *p* < 0.001) and the presence of sleep disorders (OR: 1.85, 95% CI [1.16, 2.94], *p* < 0.05) emerged as risk factors for CFD. Regarding work types, patients in mental work (OR: 0.12, 95% CI [0.04, 0.37], *p* < 0.001) and those engaged in both mental and manual work (OR: 0.5, 95% CI [0.29, 0.86], *p* < 0.05) displayed lower risk. Moreover, moderate-intensity physical activity (OR: 0.53, 95% CI [0.32, 0.87], *p* < 0.05) and vigorous-intensity physical activity (OR: 0.26, 95% CI [0.12, 0.58], *p* < 0.05), as well as the use of ACEI/ARB (OR: 0.61, 95% CI [0.39, 0.95], *p* < 0.05) and beta-blockers (OR: 0.48, 95% CI [0.25, 0.93], *p* < 0.05), were found to have a protective effect against the development of CFD.

**Table 5:** Binary logistic regression analysis of the influence of lifestyle on CFD

Research Variables	*P*-value	Univariate analysis OR [95%CI]	*P*-value	Multivariate analysis OR [95%CI]
Sex (Female)	0.904	1.02[0.7,1.49]	0.333	0.79[0.5,1.27]
Age(≥ 65)	< 0.001^*^	15.95[6.92,36.75]	< 0.001^*^	8.68[3.64,20.72]
**Educational level**				
Primary school or below		1.00		1.00
Junior high school or senior high school	0.025^*^	0.64[0.44,0.95]	0.907	1.03[0.65,1.62]
University or above	0.153	0.49[0.19,1.3]	0.621	1.33[0.43,4.1]
Married	0.123	0.46[0.17,1.23]	0.348	0.58[0.19,1.8]
**Type of work**				
Manual labor		1.00		1.00
Mental labor	< 0.001^*^	0.12[0.04,0.34]	< 0.001^*^	0.12[0.04,0.37]
Both manual and brain labor	< 0.001^*^	0.4[0.25,0.63]	0.012^*^	0.5[0.29,0.86]
Obesity	0.533	0.75[0.31,1.83]	0.579	0.75[0.28,2.05]
Physical activity level				
Light		1.00		1.00
Moderate	0.001^*^	0.49[0.32,0.75]	0.011*	0.53[0.32,0.87]
Vigorous	< 0.001^*^	0.15[0.07,0.3]	0.001*	0.26[0.12,0.58]
Sleep Disorders	< 0.001^*^	2.21[1.5,3.25]	0.01*	1.85[1.16,2.94]
Smoking	0.043^*^	0.51[0.26,0.98]	0.28	0.63[0.27,1.46]
Drinking	0.202	0.7[0.41,1.21]	0.789	0.91[0.45,1.84]
ACEI/ARBs use	0.006*	0.59[0.4,0.86]	0.029*	0.61[0.39,0.95]
Beta-blockers use	0.043*	0.57[0.33,0.98]	0.03*	0.48[0.25,0.93]
CCBs use	0.772	1.06[0.72,1.56]	0.608	1.14[0.7,1.85]
Diuretics use	0.905	0.97[0.6,1.58]	0.383	0.77[0.43,1.39]

Multivariate regression analyses: Sex, Age, Educational level, Marital status, Type of work, Obesity, Physical activity level, Sleep Disorders, Smoking, Drinking, ACEI/ARBs use, Beta-blockers use, CCBs use, Diuretics use

^*^Statistically significant value (*p* < 0.05)

### Decision tree model of the influence of lifestyle on CFD

The data were checked for accuracy and assignment of values to the research variables (Table 6). The variables that showed statistical significance in the univariate analysis were chosen for the decision tree analysis (Table 5). These variables included physical activity level, type of work, and sleep disorder, which were selected for each decision tree model node. The root node variable used was physical activity level.

**Table 6:** CFD Studies variable assignment

Research Variables	Assignment Situation
Cognitive function decline	No = 0, Yes = 1
Sex	Male = 1, Female = 2
Age	< 65 = 1, ≥ 65 = 2
Educational level	Primary school or below = 1, Junior high school, or senior high school = 2, University or above = 3
Marital status	Unmarried, divorced or widowed = 0, Married = 1
Type of work	Manual labor = 1, Mental labor = 2, Both manual and brain labor = 3
Obesity	No = 0, Yes = 1
Physical activity level	Light = 1, Moderate = 2, Vigorous = 3
Sleep Disorders	No = 0, Yes = 1
Smoking	No = 0, Yes = 1
Drinking	No = 0, Yes = 1
ACEI/ARBs use	No = 0, Yes = 1
Beta-blockers use	No = 0, Yes = 1
CCBs use	No = 0, Yes = 1
Diuretics use	No = 0, Yes = 1

**Fig. 2:**
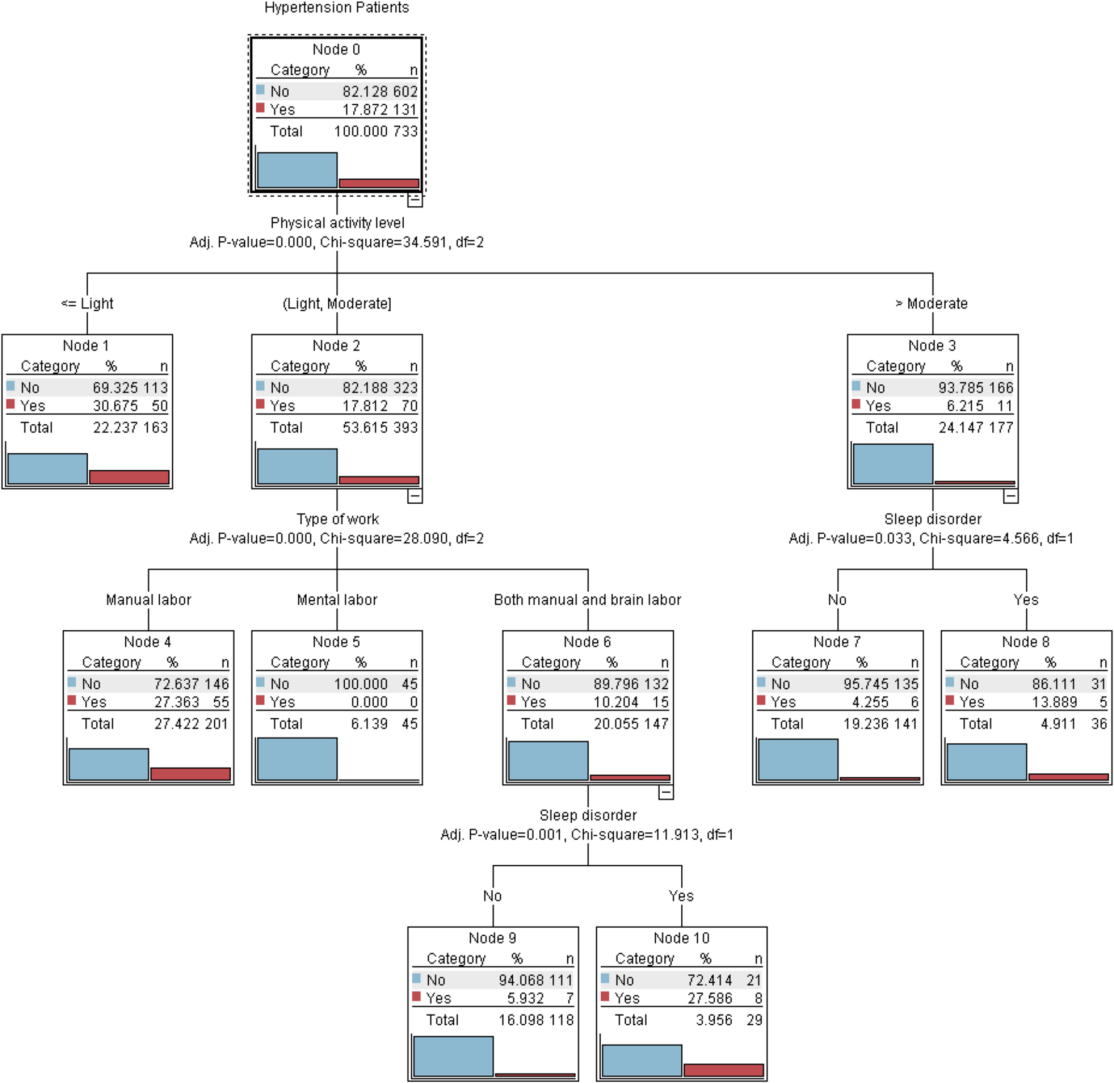
The decision tree model illustrates the impact of lifestyles on CFD and has been generated by the software using ten cross-validations. The boxes are numbered sequentially, with the blue icon representing NCI and the red icon representing CFD. Each box has a specific percentage and number of examples. The influence factor of the following classification is indicated below each box, while the corresponding category of influence factor is located at the top of each box

The results showed that the probability of CFD in hypertensive patients with moderate above physical activity was 6.215% (11 cases), lower than those with light to moderate physical activity17.812% (70 cases), and light and below physical activity was 30.675% (50 cases). Among the patients with moderate above physical activity and sleep disorders, the probability of CFD of those with sleep disorders was 13.889% (5 cases), higher than that of those without sleep disorders at 4.255% (6 cases). Among the patients with light to moderate physical activity, the probability of CFD among the hypertension patients with manual work was 27.363% (55 cases), which was higher than those with mental work (0%; 0 cases) and both manual and mental work (10.204%; 15 cases). In addition, among the hypertension patients with light to moderate physical activity and both manual and mental labor, the probability of CFD among hypertension patients with sleep disorders was 27.586% (8 cases), higher than those without sleep disorders (5.932%; 7 cases) (Fig. 2).

### The importance of prediction variables in the decision tree model

**Fig. 3:**
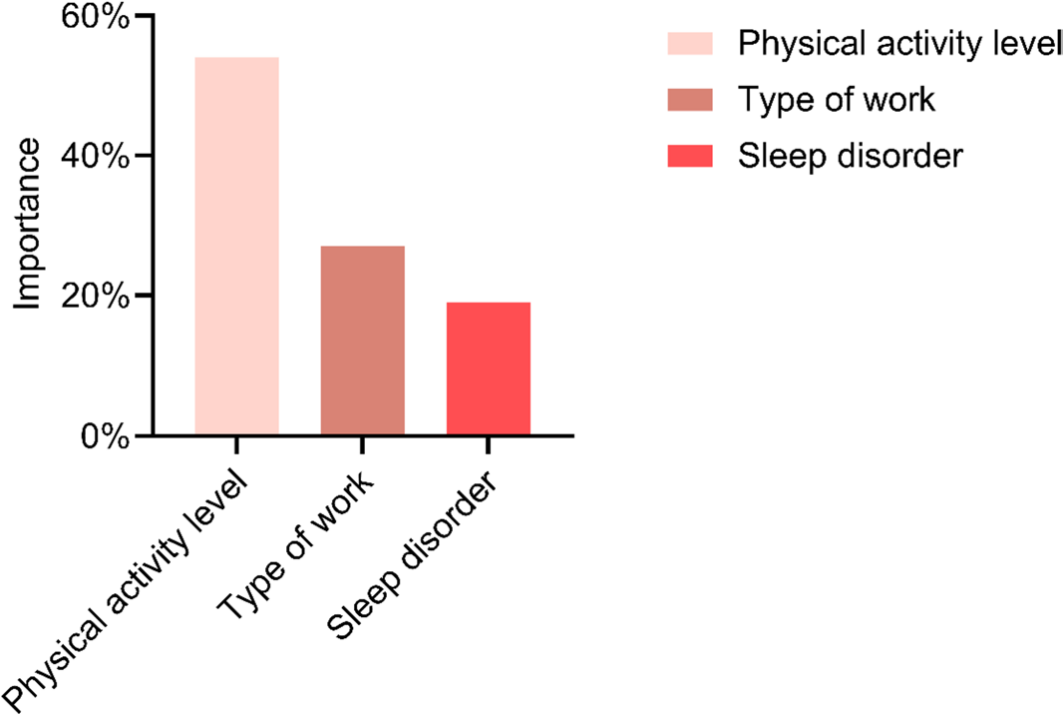
The significance of predictive variables in the decision tree model is a crucial aspect to consider. We included work type, sleep disorders, and physical activity levels in the decision tree model to assess their impact of CFD in hypertensive patients. The figure illustrates the relative importance of each influencing factor in predicting the outcome within the decision tree model. Notably, physical activity level is the root node variable deemed the most critical predictive variable

The importance of each node prediction variable to the construction of the model was different in the decision tree model. In contrast, the importance of physical activity level as the root node variable was 54%. In the leaf nodes, the importance of the type of work, and sleep disorder, were 27% and 19%, respectively (Fig. 3).

### Prediction of ROC curve of CFD Occurrence by decision tree model

**Fig. 4:**
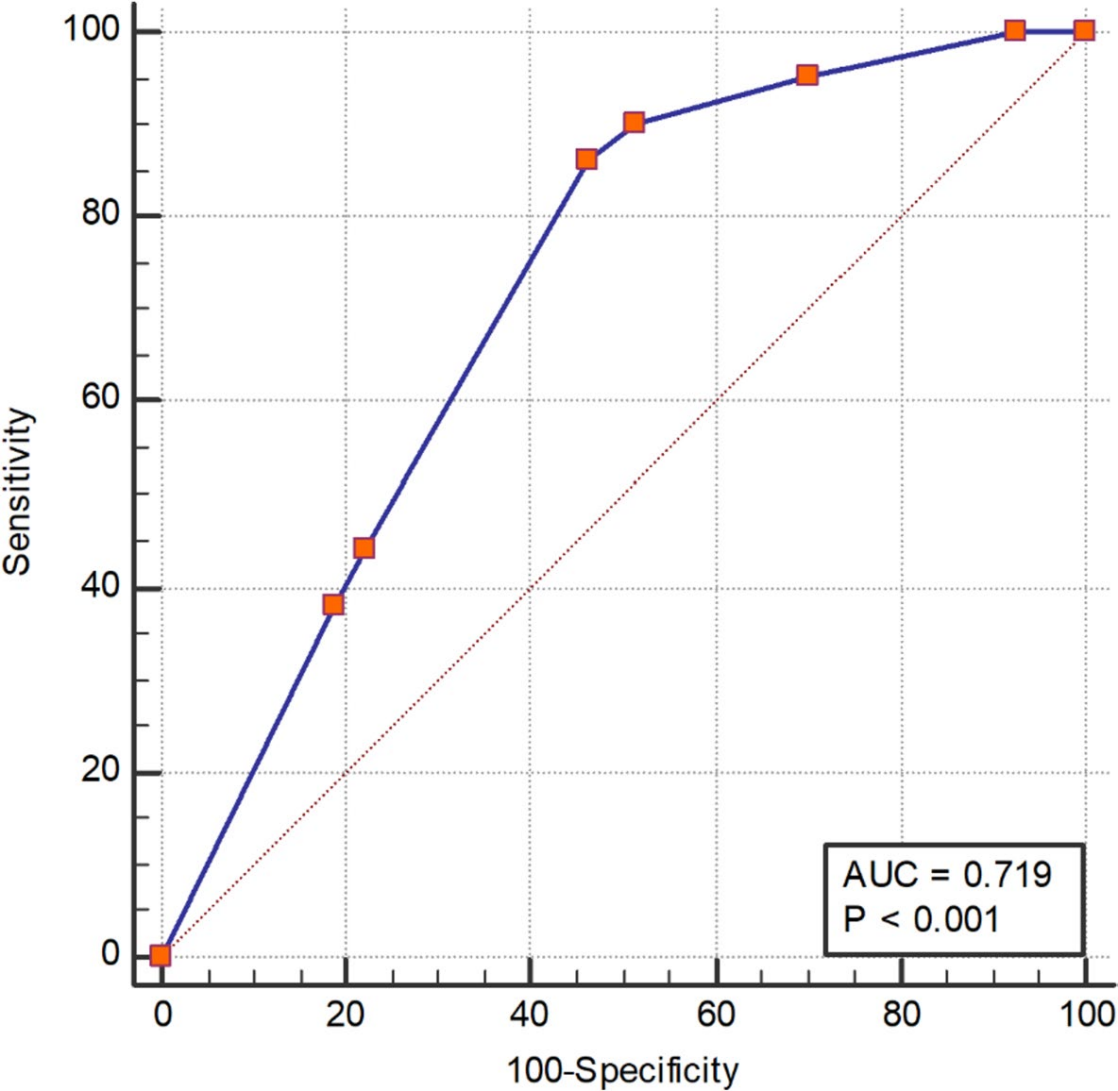
We utilized the decision tree prediction probability model to forecast the ROC curve of CFD. The dotted lines in the figure demonstrate the predictive capacity of the decision tree model in acknowledging the incidence of CFD. The AUC was 0.72, *P* < 0.001

Taking the prediction of the decision tree model as the test variable and the actual CFD data as the state variable to draw the ROC curve, it was concluded that the AUC of the decision tree model for predicting the occurrence of CFD was 0.72 (95%CI: 0.68 to 0.76, *p* < 0.001), and the sensitivity was 0.86(95%CI: 0.79 to 0.92), the specificity was 0.54(95%CI: 0.49 to 0.58). + CUI: 0.25, -CUI:0.51. This shows that the prediction effect of the decision tree model was medium (Fig. 4).

## Discussion

In this hypertension patient-focused study, we explored the connection between diverse lifestyles and cognitive decline. Our logistic regression analysis revealed that advanced age and sleep disorders pose risks for CFD. Conversely, individuals with higher education levels, those engaged in mental or both mental and manual occupations, moderate and high intensity physical activity, and users of ACEI/ARBs and Beta-blockers exhibit protective factors against CFD.

Numerous studies have delved into the positive impact of physical activity on cognitive function among adults. A meta-analysis of 15 prospective cohort studies underscored the protective influence of vigorous exercise against cognitive decline (hazard ratio 0.62, 95% CI 0.54–0.70) [39]. Furthermore, recent research conducted by Finnish and Swedish scholars demonstrated that low to moderate levels of physical activity in midlife correlated with an elevated risk of dementia when contrasted with high activity levels. Sustained high physical activity or its escalation from midlife were associated with reduced dementia risk [40].

The outcomes of our cross-sectional study align with prior research on the link between physical activity levels and cognitive function. They underline the importance of physical activity in postponing cognitive decline in hypertensive individuals. Among the potential mechanisms, several are widely recognized. Physical activity enhances the management of cardiovascular risk factors, augments cerebral blood flow and brain tissue oxygenation, and stimulates the production of neurotransmitters, neurotrophic factors, and brain-derived neurotrophic factor (BDNF), fostering neurogenesis and synaptogenesis, crucial elements in cognitive function [41–44]. While exercise interventions have proven beneficial to cognitive function, further research is necessary to elucidate the molecular mechanisms underpinning the relationship between physical activity and the risk factors for cognitive decline.

During moderate or higher intensity physical activity, we observed that hypertensive patients with sleep disorders were more prone to developing CFD. Prior research indicates that sleep disorders can impact cognitive function [45]. It is worth mentioning that 32.2% of the hypertensive participants in this study experienced poor sleep quality.

In a substantial cohort study involving older Chinese adults [46], lower habitual sleep efficiency was linked to a higher risk of memory impairment and overall cognitive decline. The impact of sleep disorders on cognitive function might be attributed to substantial reductions in brain volume in specific regions such as the bilateral hippocampus, superior parietal lobule, left amygdala, and cortical and subcortical regions following sleep deprivation [47]. Additionally, sleep deprivation hampers adult hippocampal neurogenesis [48], leading to neuron loss, decreased neuronal regeneration [49], and provoking excessive activation of hippocampal microglia [50], ultimately contributing to cognitive decline.

While the logistic regression model effectively calculates specific odds ratios associated with different influencing factors and demonstrates the relationship between CFD in hypertensive patients and these factors, it fails to illustrate interactions between variables and lacks intuitive predictions. Decision trees, on the other hand, excel in showcasing these interactions. In our study, we incorporated physical activity level, work type, and sleep disorders into the decision tree model. Among them, physical activity level emerged as the primary influencing factor at the first level of the decision tree, underscoring its pivotal role in CFD among hypertensive patients. The subsequent levels of the decision tree elucidate the intricate interplay between these factors.

A previous study conducted in China revealed that individuals involved in manual labor were more susceptible to cognitive decline [51]. In our current trial, we observed a similar trend: among hypertensive patients engaging in light to moderate physical activity, the decision tree analysis indicated that those employed in manual labor were inclined towards cognitive decline. This finding implies that engaging in physical labor may contributes to cognitive decline. Conversely, another study demonstrated that older adults engaged in mentally stimulating work tend to exhibit active thinking and superior cognitive functions, including understanding, discrimination, and reasoning, making them less susceptible to CFD [52]. However, the precise mechanisms through which type of work impacts cognitive function remain unclear. Beatriz E. Alvarado suggests that manual workers might be more vulnerable to cognitive impairment, potentially due to their lower levels of education [53].

In our study, we observed that gender did not exhibit an association with the incidence of CFD in hypertensive patients, which contrasts with some prior findings. Other studies have reported varying prevalence rates of CFD in both men [54] and women [55]. The higher prevalence of CFD in women may be attributed to hormonal disparities between genders [56]. Estrogen exposure is known to influence brain aging, impacting overall cognitive function and language attention [57]. Reduced estrogen levels in postmenopausal women can result in partial impairment of cognitive functions, such as verbal memory, reasoning, and alertness [58].

The above results indicate that the relationship between gender and prevalence of hypertensive CFD remains a topic of debate. We still need to conduct further research in this area.

The decision tree model can present the impact and likelihood of each factor incorporated in the model on CFD clearly and intuitively, allowing for easy comprehension along each primary line with a high level of readability. The decision tree model can present the impact and likelihood of each factor incorporated in the model on CFD clearly and intuitively, allowing for easy comprehension along each primary line with a high level of readability. The AUC of the ROC curve for CFD forecasted by the decision tree model is 0.72[95% CI: 0.68 to 0.76],although its predictive power is general [59], we believe that 70% is considered clinically useful [60].

We acknowledge the limitations of the current research. First, the dataset used in this study is based on cross-sectional collection, making it arduous to establish a potential causal relationship. Secondly, gender may influence cognitive ability, and the study failed to implement a gender-stratified follow-up design due to the limited sample size. Additionally, we should introduce a tool for assessing quality of life to evaluate the daily experiences of patients. Furthermore, some data comes from self-reports collected through questionnaires, which could result in bias. Third, the sensitivity and specificity of MMSE in identifying cognitive function decline are limited. In the future, further confirmation is required by combining MMSE with other cognitive function assessment scales, such as the Montreal Cognitive Assessment Scale [61, 62]. Fourth, this study was carried out on patients with essential hypertension, and the outcomes may need to be more generalizable to other populations. Still, hypertension is a notable risk factor for cognitive decline, and this population requires more attention [63].

Nevertheless, the current study has several advantages. Firstly, this study showed the practicability of employing decision tree models to anticipate mild cognitive dysfunction among hypertensive patients. Secondly, the data collection from multiple medical centers also curbs deviations to some extent and augments the dependability and adaptability of the decision tree model.

Despite some limitations, this study offers a new perspective on predicting cognitive function decline in hypertension. However, further prospective longitudinal cohort studies are encouraged to confirm the predictive power of the decision tree model.

## Conclusion

We discovered an association between hypertension and various lifestyle factors, including crucial predictors like physical activity levels, in patients with CFD. Other factors, such as work type and sleep disorder, can also predict the occurrence of CFD in patients with hypertension. In this study, the interaction between these factors was observed to affect the occurrence of hypertensive CFD patients further. Therefore, personalized lifestyle interventions may be recommended to hypertensive patients to reduce the risk of hypertensive CFD. Future studies will involve the integration of decision trees and machine learning algorithms to analyze clinical data and develop an optimal model.

## Data Availability

The datasets used and/or analyzed during the current study are available from the corresponding author on reasonable request.

## Abbreviations

NCI: No cognitive impairment
CFD: Cognitive function decline
BMI: Body mass index
MMSE: Mini-mental state examination
IPAQ: International Physical Activity Questionnaire
RAD: Right atrial diameter
LAD: Left atrial diameter
RVD: Right ventricular diameter
LVD: Left ventricular diameter
ACEI: Angiotensin converting enzyme inhibitors
ARB: Angiotensin receptor blocker
CCBs: Calcium channel blockers
SBP: Systolic blood pressure
DBP: Diastolic blood pressure
LAP: Lipid accumulation product
FBG: Fasting blood glucose
TG: Triglyceride
TC: Total cholesterol
LDL-C: Low-density lipoprotein cholesterol
SCr: Serum creatinine
PSQI: Pittsburgh sleep quality index
